# Beyond the typodont: deliberate practice, experiential learning, and feedback in postgraduate orthodontic education

**DOI:** 10.3389/fmed.2026.1890078

**Published:** 2026-09-01

**Authors:** Ali Borzabadi-Farahani

**Affiliations:** Department of Orthodontics, School of Dentistry, Cardiff University, Cardiff, United Kingdom

**Keywords:** competency-based education, feedback, orthodontic education, orthodontics, postgraduate training, workplace-based assessment

## Abstract

**Background:**

Postgraduate orthodontic education has evolved substantially, but marked global variation remains in program structure, assessment, and graduate preparedness. Feedback is central to clinical training, yet evidence specific to orthodontic education remains limited.

**Objective:**

Guided by Bordage’s educational conceptual frameworks, this review explores contemporary postgraduate orthodontic education, focusing on instructional approaches, workplace-based assessments (WBAs), and trainee perceptions of feedback through the lenses of Miller’s Pyramid of Clinical Competence, Ericsson’s Deliberate Practice theory, Kolb’s Experiential Learning, and van de Ridder’s feedback models.

**Methods:**

A narrative review was conducted using PubMed, Scopus, and Google Scholar for studies published up to 15 June 2026, with an updated search conducted prior to publication of the final version. Evidence (*n* = 88) was synthesized thematically across five domains.

**Results:**

International guidelines recommend at least 36 months of full-time orthodontic training with extensive supervised clinical experience, although substantial variation exists internationally. WBAs with timely feedback provide authentic assessment of clinical performance but are resource-intensive and influenced by assessor variability. Problem- and scenario-based learning support experiential learning and strengthen clinical reasoning beyond passive knowledge acquisition. Technology-enhanced approaches, including simulation, virtual and augmented reality, haptic systems, and flipped classroom models, may support learning and deliberate practice but are not intended to replace supervised patient care. Trainees consistently value feedback that is specific, timely, and delivered by engaged supervisors.

**Conclusion:**

Clinical competence develops through repeated supervised practice supported by meaningful feedback and principles of deliberate practice. Emerging technologies may enhance training but cannot substitute for direct clinical experience. Longitudinal research is needed to assess their impact on long-term competence and patient outcomes.

## 1 Introduction

Orthodontics is the dental specialty concerned with the diagnosis and treatment of anomalies of the dentition, craniofacial growth, and occlusion. Although the specialty is little more than a century old, advances in biomechanics, three-dimensional imaging, digital technology, and aligner systems have transformed modern orthodontic practice (1). Contemporary orthodontic care requires a combination of biological knowledge, technical skill, treatment planning ability, and interdisciplinary collaboration, all of which demand rigorous postgraduate training (1).

Postgraduate orthodontic programs aim to produce specialists capable of diagnosing complex malocclusions, planning evidence-based treatment, managing interdisciplinary cases, and evaluating outcomes critically (2). To support international consistency, the World Federation of Orthodontists (WFO) introduced postgraduate education guidelines in 2009 (3) and updated them in 2023 to reflect developments such as digital orthodontics, artificial intelligence, teledentistry, and contemporary clinical workflows (2). These guidelines are intended to provide a global benchmark for specialist training.

Despite these efforts, orthodontic education remains highly variable internationally (4). Differences exist in program duration, clinical exposure, assessment systems, research expectations, and educational philosophy. Some programs emphasize clinical workload and patient numbers, while others prioritize research and academic development (4). Similar variability has also been reported at undergraduate level, where orthodontic curricula differ substantially in learning outcomes, competency expectations, and teaching methods (5).

Feedback is a fundamental element of postgraduate clinical education. Since its wider incorporation into medical education in the 1980s, feedback has become recognized as an essential mechanism for improving performance, identifying deficiencies, and supporting reflective practice (6). In dentistry and medicine, effective feedback is associated with improved clinical competence and better patient care (6). However, there remains limited orthodontic-specific literature exploring how feedback is delivered and perceived within postgraduate training environments (6).

**Figure 1:**
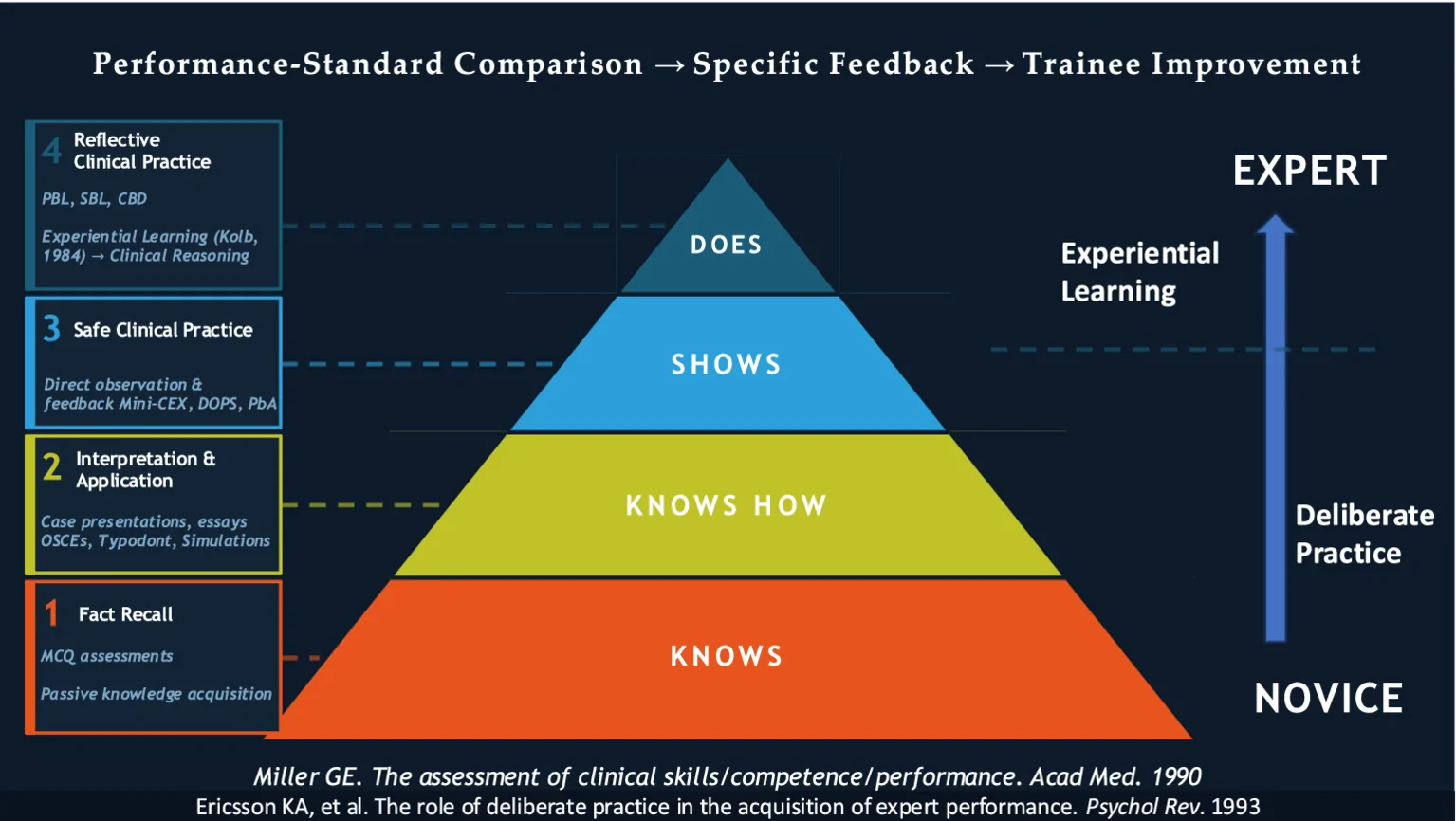
Contemporary orthodontics teaching is mainly organized around Miller’s Pyramid of Clinical Competence, Ericsson’s theory of expertise development, which emphasizes deliberate, varied practice accompanied by regular feedback, Kolb’s experiential learning theory, and van de Ridder’s feedback model as guiding conceptual frameworks (8–12, 77, 78). Deliberate practice involves educators intentionally designing learning activities (ie, Wire bending, bracket bonding) and providing immediate feedback (9, 10). This enables learners to incorporate feedback into subsequent performance, refine their clinical skills and progressively improve their competence toward achieving predefined learning outcomes. Kolb’s Experiential Learning Theory (11) proposes that learning occurs through a four-stage cycle in which experience is transformed into knowledge: concrete experience, reflective observation, abstract conceptualisation, and active experimentation. Experiential learning underpins scenario- or problem-based learning (PBL), in which the teacher creates the problem (e.g., an actor simulating an orthodontic complication) and facilitates the learning process rather than delivering content knowledge directly. PBL is most effective once learners have developed a foundational level of knowledge and understanding of the subject, and it helps them develop problem-solving, leadership, and critical thinking skills, alongside the ability to work collaboratively within a team (8–12, 77, 78).

This narrative review synthesizes the available evidence relating to postgraduate orthodontic education, instructional strategies, assessment methods, and feedback practices. Emphasis is placed on program structure, competency frameworks, workplace-based assessments, technology-enhanced learning, problem-based and scenario-based learning, and trainee perceptions of feedback. Guided by Bordage’s (7) argument that educational research should be explicit about the ‘conceptual frameworks’ used to interpret findings, this review is organized around four complementary theoretical lenses (Figure 1). First, Miller’s Pyramid of Clinical Competence (8) provides a hierarchical model for mapping educational interventions to levels of performance, allowing structured comparison of didactic, simulated, and authentic clinical teaching methods. Second, Ericsson’s theory of deliberate practice (9, 10) illuminates why repeated, supervised, feedback-rich encounters such as in Mini-CEX and WBAs, are prerequisite for skill acquisition, and why clinical judgment lags behind procedural competence in early training. Third, Kolb’s experiential learning theory (11), and fourth, the van de Ridder et al.’s (12) conceptualisation of feedback as a social, dialogic, and information-rich process grounds the analysis of trainee perceptions and identifies what makes feedback effective versus ineffective in specialist orthodontic training. Together, these frameworks highlight different variables and outcomes, and their inter-relatedness, while acknowledging that any single framework offers only a partial view of the educational landscape (7).

## 2 Methodology

This study was conducted as a narrative review examining contemporary approaches to postgraduate orthodontic education, with particular emphasis on program structure, curriculum design, instructional strategies, workplace-based assessments, technology-enhanced learning, and feedback practices. A narrative review methodology was selected because of the broad and multidisciplinary nature of the topic, which incorporates educational theory, clinical training frameworks, assessment methods, and trainee perceptions across different international educational systems.

A comprehensive literature search was performed using the electronic databases PubMed, Scopus, and Google Scholar. The search included articles published up to 15 June 2026, with an updated search prior to publication of the final version to capture both foundational educational literature and contemporary developments in orthodontic training. Additional manual searching of reference lists from included articles was also undertaken to identify relevant publications not retrieved during the electronic search.

### 2.1 Population–Concept–Context (PCC) framework

The following Population–Concept–Context (PCC) framework was used to guide this review:

*Population*: Postgraduate/specialist trainees in orthodontics (residents, postgraduate dental trainees, specialty registrars).

*Concept*: Educational and instructional strategies relevant to postgraduate orthodontic training, including curriculum design, technology-enhanced learning (three-dimensional simulation, augmented reality/virtual reality, haptics), problem-based and scenario-based learning, flipped classroom approaches, traditional didactic and apprenticeship-based teaching models, and workplace-based assessment tools (Mini-CEX, OSCE, WBA) used as comparators or companions to didactic methods. The concept also encompassed clinical competence acquisition (per Miller’s Pyramid), skill development (per Ericsson’s deliberate practice), the quality and trainee perception of feedback (per van de Ridder et al.), and reflective learning outcomes (per Kolb’s Experiential Learning Theory).

*Context*: Postgraduate orthodontic training programmes delivered within university dental schools, teaching hospitals, and affiliated clinical training sites, encompassing both formal curricular settings and workplace-based clinical training environments.

Search terms included combinations of keywords and Medical Subject Headings (MeSH) related to postgraduate orthodontic education and clinical teaching. The primary search terms included: “orthodontic education”, “orthodontic residency”, “postgraduate orthodontic training”, “orthodontic postgraduate education”, “orthodontic curriculum”, “clinical teaching”, “clinical competence”, “teaching strategies”, “skill development”, “didactic teaching”, “three-dimensional simulation”, “augmented reality”, “virtual reality”, “haptic technology”, “educational interventions”, “feedback”, “workplace-based assessment”, “Mini-CEX”, “OSCE”, “scenario-based learning”, “problem-based learning”, “technology-enhanced learning”, “virtual learning” and “flipped classroom.” Boolean operators (“AND,” “OR”) were used to refine searches and maximise retrieval of relevant literature.

The review included peer-reviewed journal articles (case series, randomized clinical trials), systematic reviews, educational studies, qualitative studies, consensus statements, guideline documents, and policy papers related to postgraduate orthodontic education. Where available, priority was given to secondary evidence, including systematic reviews and scoping reviews. Publications focusing exclusively on undergraduate dental education without relevance to postgraduate training were excluded unless they provided important contextual comparisons. Editorials, opinion articles without supporting evidence, and non-English language publications without accessible translations were also excluded. In addition to published literature, official guideline documents from the World Federation of Orthodontists, the Network of Erasmus-Based European Orthodontic Postgraduate Programs, and the Royal Colleges were reviewed to provide insight into international educational standards and competency frameworks. These documents were included because they represent authoritative recommendations influencing orthodontic training globally.

### 2.2 Literature search

The following PubMed search was used:

(“orthodontic education”[tiab] OR “orthodontic residency”[tiab] OR “postgraduate orthodontic training”[tiab] OR “orthodontic postgraduate education”[tiab] OR “orthodontic curriculum”[tiab] OR “Orthodontics/education”[Mesh] OR “Education, Dental, Graduate”[Mesh]) AND (“clinical teaching”[tiab] OR “clinical competence”[tiab] OR “Clinical Competence”[Mesh] OR “teaching strategies”[tiab] OR “skill development”[tiab] OR “three-dimensional simulation”[tiab] OR “3D simulation”[tiab] OR “augmented reality”[tiab] OR “Augmented Reality”[Mesh] OR “virtual reality”[tiab] OR “Virtual Reality”[Mesh] OR “haptic technology”[tiab] OR “haptic feedback”[tiab] OR “educational interventions”[tiab] OR “problem-based learning”[tiab] OR “Problem-Based Learning”[Mesh] OR “scenario-based learning”[tiab] OR “technology-enhanced learning”[tiab] OR “virtual learning”[tiab] OR “flipped classroom”[tiab]) AND (“didactic teaching”[tiab] OR “apprenticeship”[tiab] OR “feedback”[tiab] OR “Feedback”[Mesh] OR “workplace-based assessment”[tiab] OR “work-based assessment”[tiab] OR “Mini-CEX”[tiab] OR “mini clinical evaluation exercise”[tiab] OR “OSCE”[tiab] OR “objective structured clinical examination”[tiab]) AND (“1900/01/01”[Date - Publication]: “2026/06/15”[Date - Publication]). This search identified 48 records.

The following Scopus search was used:

TITLE-ABS-KEY ((“orthodontic education” OR “orthodontic residency” OR “postgraduate orthodontic training” OR “orthodontic postgraduate education” OR “orthodontic curriculum”) AND (“clinical teaching” OR “clinical competence” OR “teaching strategies” OR “skill development” OR “three-dimensional simulation” OR “3D simulation” OR “augmented reality” OR “virtual reality” OR “haptic technology” OR “haptic feedback” OR “educational interventions” OR “problem-based learning” OR “scenario-based learning” OR “technology-enhanced learning” OR “virtual learning” OR “flipped classroom”) AND (“didactic teaching” OR “apprenticeship” OR “feedback” OR “workplace-based assessment” OR “work-based assessment” OR “Mini-CEX” OR “mini clinical evaluation exercise” OR “OSCE” OR “objective structured clinical examination”)) AND PUBYEAR < 2027. This search identified 11 records.

After adding relevant material from PubMed, Scopus, and Google Scholar, reviewing the reference lists of relevant articles, guidelines, and addition of early online publications, a total of 88 articles and documents were identified and used for this narrative review.

Following identification of eligible sources, the literature was reviewed and synthesized thematically. Thematic categories were developed iteratively following repeated reading of the included literature. Five principal domains were identified:

- Program structure and international standards,
- Curriculum content and competency frameworks,
- Instructional strategies and teaching delivery methods,
- Structured clinical and workplace-based assessments, Problem- and scenario-based learning, Technology-enhanced approaches, and
- Feedback practices in orthodontic education.

Evidence within each domain was summarized descriptively, with attention given to similarities, differences, advantages, limitations, and emerging educational trends.

The narrative review approach allowed integration of diverse forms of evidence and facilitated critical discussion of educational practices, although limitations inherent to narrative reviews, including potential selection bias and lack of formal quality appraisal, are acknowledged.

## 3 Results and discussion

### 3.1 Program structure and global standards

The WFO 2023 guidelines provide the most comprehensive international framework for postgraduate orthodontic education (2). They recommend a minimum of 36 months of full-time specialty training, allowing sufficient time for clinical case completion, interdisciplinary management, research activity, and retention assessment (2). Applicants are ideally expected to have completed at least two years of general dental practice prior to entry (2).

The guidelines recommend approximately 24 h of supervised clinical activity per week and assignment of at least 30 new patients to each trainee, with 50 cases strongly encouraged where possible (2). In addition, programs should include regular case presentations, journal clubs, and research training (2). Faculty supervision is emphasized, with a recommended clinical staff-to-resident ratio of at least 1:6 (2). For the UK 3 year orthodontic specialist training, suggested case-mix guidance recommends trainees treat 100–140 patients with a broad spectrum of malocclusions, with case transfers capped at no more than 30% of the trainee’s overall caseload (85).

Despite these recommendations, considerable variation persists internationally (4). A systematic review by Baxmann et al. (4) demonstrated substantial heterogeneity in program structure, clinical exposure, research expectations, and assessment systems across Europe and North America. Some programs provide extensive clinical experience, whereas others focus more heavily on research and academic output (4). Such variability may contribute to differences in graduate preparedness and clinical competence.

An Italian study involving 312 dentists highlighted the influence of postgraduate specialist training on clinical practice (1). Orthodontic specialists were significantly more likely than non-specialists to practise exclusively in orthodontics, work within interdisciplinary teams, and use straight-wire mechanics (1). These findings suggest that formal specialist education influences not only technical competence but also professional practice patterns and treatment philosophy (1).

Alongside the WFO framework, Europe has developed its own coordinated educational standards through the Network of Erasmus-Based European Orthodontic Postgraduate Programs (NEBEOP) (13). Originating from the Erasmus Project initiated in 1989, NEBEOP established recommendations regarding program duration, clinical exposure, supervision, research activity, and final assessment (13). The NEBEOP guidelines require full-time university-based training with at least 2000 clinical hours and approximately 50 supervised cases (13). Students must complete a research project and present documented treated cases before an examination panel (13). These guidelines have significantly influenced international orthodontic education and contributed to later revisions of the WFO standards (13).

There is variation in the level of competency expected for some aspects of orthodontic training, such as combined orthodontic-surgical treatment of dentofacial deformity. In the USA, graduates of orthodontic residency programs must be “competent to treat and manage major dentofacial abnormalities and coordinate care with oral and maxillofacial surgeons and other healthcare providers” (86). However, a distinct post-residency tier has emerged in the form of12-month Craniofacial and Special Care Orthodontics Fellowships. These fellowships provide “advanced education and training in focused areas such as “cleft lip/palate patient care, syndromic patient care, orthognathic surgery, craniofacial surgery, and special care orthodontics” (87).

The UK’s GDC 2024 standard (38) sets a comparatively modest baseline for regular orthodontic specialists, requiring only an “understanding” of craniofacial disorders as part of general specialist orthodontic training. Trainees are taught to “recognise and refer” complex surgical or facial deformity cases rather than manage them independently; full competency in this area is instead achieved through an additional two-year hospital orthodontic consultant training pathway.

The WFO 2023 guidelines (2), by contrast, keep this content within the single 36-month generalist track. All graduates are expected “to collaborate in the interdisciplinary treatment” of complex cases, including syndromes, craniofacial anomalies, and orthognathic surgery care, without any additional training tier, minimum duration, or separately accredited pathway attached to this expectation (2).

### 3.2 Curriculum content and competency frameworks

The WFO curriculum framework encompasses biomedical sciences, orthodontic theory, clinical orthodontics, research methodology, and interdisciplinary care (2). Core subjects include craniofacial growth and development, genetics, biomechanics, cephalometrics, orthodontic materials, orthognathic surgery, and practice management (2). Trainees are also expected to complete original research and present fully documented treated cases (2).

The 2023 WFO revision expanded the curriculum to include digital orthodontics, artificial intelligence, teledentistry, social media management, and accelerated tooth movement techniques (2). These additions reflect the rapid technological evolution of the specialty and the growing importance of digital workflows in clinical practice.

Competency-based educational frameworks have also been explored through Delphi consensus studies. Ferrer et al. (14) identified key curriculum domains including diagnostic records, malocclusion classification, treatment planning, biomechanics, and orthodontic appliances. The study demonstrated that postgraduate orthodontic education requires substantially higher cognitive competency levels than undergraduate dental education (14).

At undergraduate level, orthodontic teaching often focuses on diagnosis, referral, and recognition of treatment need rather than comprehensive treatment delivery (5). Studies consistently show that dental graduates feel underprepared in areas such as appliance adjustment, orthodontic diagnosis, and treatment planning (5). Consequently, postgraduate orthodontic programs must build substantially upon a limited undergraduate foundation.

### 3.3 Instructional strategies in postgraduate orthodontic education

#### 3.3.1 Didactic teaching and evidence-based learning

Traditional didactic teaching continues to provide essential theoretical foundations in orthodontic education (2). Topics such as biomechanics, craniofacial biology, biostatistics, and research methodology remain integral components of postgraduate curriculum (2). Evidence-based practice is increasingly emphasized within residency programs (2) and orthodontic trainees are expected to critically evaluate literature, interpret statistical analyses, and conduct original research (2). However, several studies have identified deficiencies in postgraduate understanding of biostatistics and evidence appraisal (4, 15, 16). These findings suggest that research training and statistical education require stronger integration within orthodontic curricula.

#### 3.3.2 Learning styles or preferences in orthodontic trainees

Hughes et al. (17) surveyed 261 orthodontic residents in the US and Canada utilizing the Index of Learning Styles, which assesses student learning preferences in four dimensions (active/reflective, sensing/intuitive, visual/verbal, and sequential/global) (17, 18). They found that most participants demonstrated a preference for visual learning (72.4%), with a marked preference for sensing (52.5%) and sequential learning styles. Most residents were also balanced on the active/reflective scale (17). Learning preferences appear to evolve as students progress through different stages of their academic training (19). An important consideration is whether these variations in learning style significantly influence examination performance and overall academic achievement. However, previous research by Wilkinson et al. (20) demonstrated that although students exhibit diverse learning preferences, these differences have minimal impact on academic outcomes, including performance in assessments such as single best answer questions, short-answer examinations, and Objective Structured Clinical Examinations. Pashler et al. (21) reviewed the literature to evaluate the popular theory that individuals learn best when instruction is tailored to their specific “learning style,” e.g., visual, auditory. The authors concluded there is no credible scientific evidence to justify incorporating learning-style assessments into educational practice (21).

A review by Rao et al. (22) examining learning styles within orthodontic education identified only eight studies, highlighting the limited evidence currently available in this field. Consequently, there remains insufficient understanding regarding the most effective teaching approaches for both the theoretical and practical components of orthodontic training, emphasizing the need for further educational research. Nevertheless, several commonly used instructional methods are summarized below.

#### 3.3.3 Trainee learning preferences and student experience

A recent systematic review revealed that Orthodontic trainees often demonstrate strong preferences for visual and kinesthetic learning methods (4). Educational approaches incorporating videos, digital models, simulations, and case-based discussions are therefore associated with higher levels of engagement and knowledge retention (4).

Although online learning offers flexibility and convenience, many postgraduate trainees continue to prefer face-to-face teaching and supervised clinical interaction (4). Blended learning approaches combining digital resources with direct clinical teaching appear most acceptable to trainees (4). Surveys of orthodontic trainees have also identified perceived deficiencies in several contemporary treatment areas, including temporary anchorage devices, aligner therapy, lingual orthodontics, adult orthodontics, and healthcare management topics such as NHS contracts (6). These findings suggest that postgraduate curricula must continue evolving to reflect changing clinical practice demands. The COVID-19 pandemic further highlighted the importance of direct clinical experience (4). Reduced patient contact during lockdown periods negatively affected trainee confidence, procedural experience, and perceived preparedness for independent practice (4).

### 3.4 Structured clinical and workplace-based assessments, problem- and scenario-based learning, and technology-enhanced approaches

Beyond didactic instruction, postgraduate orthodontic education relies on structured methods that let trainees demonstrate and refine clinical competence under authentic conditions. This section explores simulation-based and workplace-based assessments, alongside problem- and scenario-based learning and technology-enhanced approaches, each mapping onto different levels of Miller’s Pyramid of Clinical Competence, as summarised in Table 1 and discussed below.

**Table 1:** Summary of main clinical teaching and assessment approaches used in postgraduate orthodontic education, with key advantages and limitations.

Teaching approach	Key advantages	Limitations
Didactic/Lecture-based teaching	Provides systematic theoretical foundation; efficient for large groups; well-established for biomedical and biomechanical content	Passive learning format; lower engagement and retention; does not develop clinical decision-making or procedural skills; primarily addresses the lower levels of the Miller’s Pyramid of Clinical Competence (knows, knows how)
OSCE	Assess specific clinical competencies across multiple ‘standardised’ stations; reduces examiner variability; enables repeated exposure; covers multiple competency domains simultaneously	Resource-intensive; examiner calibration required; may not fully replicate complex real-world clinical judgment; primarily address the lower levels of the Miller’s Pyramid of Clinical Competence (knows, knows how)
Mini-CEX	Assesses real patient encounters; immediate structured feedback; supports skill development over time; applicable across multiple clinical contexts; with repetition Mini-CEX organization and efficiency scores improved progressively	Dependent on assessor calibration; direct observation can induce trainee stress; variable feedback quality across supervisors; targets the ‘shows how’ level of Miller’s Pyramid of Clinical Competence; with repetition clinical judgment was the weakest and slowest-developing domain
Direct observation of procedural skills; procedure-based assessment	Evaluates technical procedural competence in authentic settings; allows repeated assessment; mirrors clinical reality; immediate feedback	Limited to observable technical skills; may not assess clinical reasoning or communication; time-consuming for supervisors
Case-based discussion	Develops clinical reasoning and evidence application; promotes reflective dialog; flexible across specialties; immediate feedback	Quality depends on supervisor engagement; may not capture procedural competence; susceptible to assessor bias
Multi-source feedback	360-degree perspective; captures professionalism and teamworking; includes patient and colleague input	Responses may reflect interpersonal relationships rather than true competence; variable response rates; limited specificity
Case/scenario-based learning	Based on ‘*Experiential learning’*; exposes trainees to predefined clinical situations; *Structured guidance supports* complex case decision-making; improves multi-disciplinary planning; increases engagement vs. didactic teaching; encourages active learning clinical reasoning/judgment; improvement in communication skills and interpersonal skills; and not merely passive knowledge acquisition.	Dependent on quality of case design; may not replicate unpredictability of real clinical encounters
Problem-based learning	Promotes self-directed learning; develops critical thinking and independent reasoning; improves integration of theory and practice; encourages ‘active learning’; improves clinical reasoning/judgment; and not merely passive knowledge acquisition.	Can generate trainee anxiety in those unfamiliar with self-directed formats; relies on group dynamics; variable tutor facilitation quality
Technology-enhanced learning	Supports self-paced study; improves retention of biomechanical concepts; increases accessibility; valuable during disruptions (e.g., COVID-19 pandemic); In ‘Flipped Classroom’ model, the conventional lecture-centered format is inverted by giving students access to instructional materials before class that encourages ‘active learning’	Reduced faculty interaction; technical limitations; variable learner engagement; cannot replace supervised direct patient care

OSCE, Objective Structured Clinical Examination; Mini-CEX, Mini-Clinical Evaluation Exercise.

#### 3.4.1 Bridging simulation and clinical competence: from typodont training to authentic assessment environment and deliberate practice

Didactic teaching and Objective Structured Clinical Examination (OSCE) (23, 24) primarily assess the lower levels of Miller’s pyramid (“knows” and “knows how”), while Mini-Clinical Evaluation Exercise (Mini-CEX) and Direct Observation of Procedural Skills (DOPS) evaluate the “shows how” level. Longitudinal Workplace-Based Assessments (WBAs) conducted in authentic clinical settings reach the “does” level of competence (8). Between these stages, simulation-based and typodont training provide an important transitional step, allowing trainees to rehearse procedural skills, including bracket bonding, wire bending, appliance fitting, diagnosis, and treatment planning, under controlled and repeatable conditions before patient contact (25, 26).

Structured clinical assessments such as OSCE and Mini-CEX are widely used in postgraduate orthodontic training to evaluate clinical competence and support constructive feedback (4). OSCEs assess specific competencies across multiple standardized stations, improving consistency and reducing variability between examiners and clinical cases (4). Repeated OSCE exposure has been associated with improvements in resident performance and confidence (4, 23). However, OSCE implementation remains resource-intensive, requiring examiner calibration and case standardization, and may not fully capture the complexity of clinical judgment in real patient care (4, 27).

In contrast, the Mini-CEX, a form of WBA, provides greater authenticity because it is conducted with real patients in clinical environments and incorporates direct observation followed by immediate feedback (4). Evidence suggests repeated Mini-CEX assessments can enhance clinical reasoning, decision-making, and communication skills, although outcomes depend on assessor calibration, consistency, and feedback quality, while direct observation may also generate stress for trainees (4, 27).

The Mini-CEX emerged in response to limitations of traditional clinical assessment approaches in medicine and dentistry. Miller’s Pyramid emphasizes that effective assessment should reach the “does” level by evaluating real-world performance rather than knowledge recall alone (8, 23, 24). Norcini and Burch (28) applied this framework to workplace-based assessment and highlighted the need for formative approaches that provide meaningful feedback opportunities. However, a 2008 study of 56 U.S. dental schools demonstrated that 62% of assessment methods remained traditional (e.g., multiple-choice examinations and laboratory practicals), whereas only 3% involved direct procedural observation, highlighting limited adoption of WBAs (29).

Norcini and colleagues (30) developed the Mini-CEX as a method of simultaneously assessing trainees’ clinical skills and offering them immediate feedback on their performance. This was a modification of the Clinical Evaluation Exercise (CEX), or traditional bedside oral examination, where a skilled clinician/educator observes a trainee’s short interaction with a patient (e.g., 15 min) and provides feedback (30–34). Studies indicated that findings from a long CEX (e.g., 2 h) were not a good predictor of trainee performance, and therefore the new format of Mini-CEX was introduced to assess performance and competencies on multiple occasions (30–34), using different patients and assessors. The revised Mini-CEX evaluates six clinical competency domains and one global rating through multiple brief observed consultations (approximately 20 min each) followed by structured feedback (30–34).

Al-Jewair and Kumar (27) assessed the Mini-CEX as a form of WBA for orthodontic residents across seven domains. Findings suggested that residents performed strongest in organization and efficiency, while ‘clinical judgement’ was consistently the weakest domain across all encounters, a finding with direct curriculum implications. Clinical performance showed a meaningful upward trend across the four encounters, suggesting that repeated direct observation combined with immediate feedback supports skill development over time (27, 35). The finding that Mini-CEX scores improved progressively across four encounters, and that clinical judgment was the weakest and slowest-developing domain, is aligned with Ericsson’s ‘deliberate practice’ theory (9, 10). Ericsson et al. (9, 10) identified task characteristics that produce expert performance: the task must motivate through real-life performance, account for pre-existing knowledge, allow repetition, provide immediate feedback, vary across contexts, and requires repetition outside a clinician’s comfort zone. Ericsson et al. (9, 10) explain that neither memorizing a clinical subject nor simply gaining extensive experience leads to expert performance; rather, it requires deliberate practice over time.

Within this context, clinical judgment lags behind procedural skills as it requires more variable, complex case exposure to build the necessary scheme and a few clinical encounters may be insufficient for some trainees. Ericsson (9, 10) specifically addresses the application of ‘deliberate practice’ in medicine, rather than mere accumulated professional experience, and it has been applied to the simulation practice, where it involves the provision of immediate feedback, time for problem-solving and evaluation, and opportunities for repeated performance to refine behavior.

#### 3.4.2 Workplace-based assessments

Workplace-Based Assessments (WBAs) evaluate trainee performance in authentic clinical settings (36) and have become increasingly integrated into postgraduate orthodontic education (28, 37). WBAs assess higher levels of Miller’s pyramid of competence, focusing on ‘what trainees actually do in clinical practice’ rather than what they can demonstrate in examinations (28, 37).

Common WBA tools include Mini-CEX, Direct Observation of Procedural Skills (DOPS), Case-based Discussion (CbD), and Multi-Source Feedback (MSF) (32). DOPS assesses technical procedures such as bracket placement or appliance fitting, while CbD evaluates clinical judgment and treatment planning (1). MSF gathers opinions from colleagues and occasionally patients to provide broader assessment of professionalism and communication (37).

WBAs offer several advantages. They encourage continuous learning, provide opportunities for immediate feedback, and allow assessment across multiple clinical contexts (28, 37). The use of multiple assessors and repeated encounters improves reliability and provides a more comprehensive evaluation of trainee performance (28, 32). The WBA tools available within UK 3-year full-time orthodontic training include (38).

- Case-based Discussion: structured dialog exploring clinical reasoning, decision-making, and evidence application across real patient cases
- Mini-Clinical Evaluation Exercise (mini-CEX): direct observation of clinical encounters with immediate structured feedback
- Consent mini-CEX: focused assessment of informed consent processes within the clinical encounter
- Direct Observation of Procedural Skills (DOPS) or Procedure-based Assessments (PbA): assessment of technical procedural competence including appliance management and clinical procedures
- Observation of Teaching (OoT): assessment of the trainee’s teaching and educational facilitation skills
- Assessment of Audit (AoA): evaluation of audit and quality improvement activity
- Multi-source Feedback (MSF): 360-degree feedback from colleagues, patients, and other members of the clinical team, assessing leadership and teamworking competencies.

However, WBAs’ implementation challenges remain significant (37). Time pressures, administrative burden, inconsistent assessor training, and concerns regarding “tick-box” assessment culture have all been reported (37, 39, 40). Orthodontics also presents unique difficulties because trainees encounter highly individualized patient scenarios rather than repetitive standard procedures (27). Consequently, WBAs should complement rather than replace high-stakes summative assessments (35).

#### 3.4.3 Problem-based and scenario-based learning

The term “conceptual learning strategies” (79) is applied more broadly within education to describe teaching methods that foster higher-order thinking skills essential to clinical reasoning, among them critical thinking, problem-solving, the retrieval and application of knowledge, and reflective judgement. Conceptual learning strategies include problem-based learning (PBL), case-based learning (CBL) (41), and scenario-based learning (SBL). PBL, CBL, and SBL are increasingly used within postgraduate orthodontic education, promote ‘deeper learning’, helping students to learn from ‘experience’ and to go through real-world examples or cognitive challenges (4, 42–45). These approaches are based on Kolb’s Experiential learning’ (11), e.g., learning through new experience, and then reflecting on it to encourage ‘active learning’, critical thinking, independent clinical reasoning, and not merely passive knowledge acquisition (4, 43).

These approaches also improved student attitudes toward subject matter with interactive or discussion classes versus straight lecturing (44, 46). SBL exposes trainees to predefined clinical situations through simulations or case scenarios, helping develop decision-making skills in complex or uncommon cases (4). Studies suggest that SBL improves preparedness for multi-disciplinary treatment planning and orthodontic emergencies (42–44). Scenario-based simulations and learning, where students engage in role-playing both as doctors and patients have been shown to improve doctor-patient communication, enhance students’ ability to manage patient expectations and emotions, and develop a more patient-centered approach (26).

PBL encourages trainees to work independently through clinical problems with minimal instructor direction, usually consisting of a small group of students and a tutor (4). Students will be presented with a ‘real-world problem’ or scenario, and they will have to work together to find a solution and answer clinical questions. This approach promotes self-directed learning and integration of theoretical knowledge with clinical practice (4). Qualitative studies report improved trainee engagement and deeper understanding when PBL methods are incorporated into orthodontic curricula (4). PBL involves minimal briefing and guidance, allowing learners to “explore tangents” whilst a CBL or SBL approach suggests structured nature and ‘increased guidance’, with guided questioning (4, 45). Compared to CBL or SBL, PBL employs continuous reflection and the lack of direction and minimal input from instructor can create stress among students unfamiliar with self-directed learning (4, 42, 45).

#### 3.4.4 Technology-enhanced learning

Orthodontic practice requires knowledge and skills of head and neck anatomy, growth and development, material science, physiology, bone biology, and mechanobiology of tooth movement (47, 81). A competent orthodontist requires skill in the cognitive, affective and psycho-motor domains (25, 47, 48). The cognitive domain covers how learners acquire knowledge and engage in thinking or reasoning (88). The affective domain relates to emotions; the attitudes, values, and interests learners hold (88). The psychomotor domain involves physical skills developed through muscle coordination and movement-based learning (88). In particular, psychomotor skills, such as wire bending and bracket positioning are at the core of needed clinical skills (47). Competency in the affective domain, such as communicating with patients and their parents are similarly important (25). Therefore, learning and practice on typodonts and simulated environment followed by clinical settings is necessary for contemporary orthodontic postgraduate programs (3). Repetitive simulation-based training improves the psychomotor abilities due to a secure, repeatable simulation practice environment (48, 49). Various forms of simulation have been reported including augmented reality (50), virtual reality (51), haptic technology (49, 52), covering aspects such as diagnosis, treatment planning, orthodontic bracket positioning, orthodontic case assessment, orthodontic procedures, facial landmark identification, and cephalometric tracing (48, 49).

Technology-enhanced learning and blended learning (53, 54) have assumed an expanding role in postgraduate orthodontic training. Multimedia tools, including recorded lectures, digital study models, and case-based simulations, have been shown to improve retention of biomechanical concepts and support self-directed study (4, 55–57). Shah and Cunningham (57) reported that integration of a virtual learning environment into a UK orthodontic program was broadly acceptable to both trainees and educators, though it required deliberate design to maintain clinical relevance. Digital learning also allows trainees to revisit complex topics independently and supports self-paced learning (4).

Clear aligner therapy relies on digital treatment simulation as a core clinical workflow, yet many orthodontists report having been underexposed (76) to this technology during postgraduate training, a finding that underscores the need for its systematic integration into orthodontic curriculum (58).

One potentially more effective application of virtual learning environments is the Flipped Classroom approach (54, 59–61). In this pedagogical model, the conventional lecture-centered format is inverted by giving students access to instructional materials before class, and therefore, the lecture and homework elements are “flipped.” (60, 62) Learners can therefore develop core knowledge independently and at their own pace through resources such as assigned readings, recorded lectures, and multimedia content. Classroom sessions are then dedicated to active learning activities, including case-based discussions and team-oriented exercises that build upon the preparatory material, thereby encouraging deeper understanding and higher-level critical thinking skills (63, 64).

A few studies of the flipped classroom model for orthodontic topics in undergraduate students revealed comparable (64) or significant improvements (65) in cognitive outcomes, knowledge retention, engagement, and student satisfaction compared to traditional lectures.

Technology-enhanced learning also presents challenges, including reduced faculty interaction, technical limitations, and variability in learner engagement (4, 66, 67). Orthodontic trainees showed a preference for a blended approach to learning where practical skills were taught in person and some theoretical aspects taught remotely (67). These findings reinforced the importance of direct patient care and supervised clinical experience in orthodontic training (4). Current evidence therefore supports the use of digital tools as supplements rather than replacements for clinical teaching (4). Finally, emerging generative artificial intelligence technologies have considerable potential to support scenario-based learning by generating authentic or near-authentic educational content (82, 84). Through experiential learning, these scenarios can foster active engagement, improve knowledge retention, and enhance clinical reasoning and decision-making skills (82, 83). However, their implementation is hindered by several barriers, including limited faculty training, inadequate technical infrastructure, and resistance among educators (83).

### 3.5 Feedback practices in orthodontic education

#### 3.5.1 Importance of feedback

Feedback is central to postgraduate clinical education and is widely recognized as essential for professional development (6, 12, 68, 69). According to Van de Ridder et al. (12) feedback is a ‘specific’ information about the comparison between a trainee’s observed performance and a standard, given with the intent to improve the trainee’s performance. Effective feedback helps trainees identify strengths, recognize deficiencies, and refine clinical performance (6). Modern educational theory increasingly views feedback as a collaborative and interactive process rather than a unidirectional transfer of information (6). Learners are expected to actively engage with feedback, reflect on performance, and implement changes in future practice (6).

Despite its recognized importance, relatively little orthodontic-specific research exists regarding feedback practices and trainee perceptions (6). Evidence from broader medical and dental education consistently demonstrates that trainees value feedback that is timely, personalized, and constructive (6, 70, 71). Overall, vague, inconsistent, or delayed feedback tends to fail, and immediate verbal feedback (6, 12) from a supervisor is preferred.

#### 3.5.2 Trainee perceptions of feedback

A national UK survey of orthodontic trainees provides important insight into feedback experiences within specialist orthodontic education (6). Almost all respondents agreed that effective feedback improved clinical performance, and most reported receiving feedback they considered useful (6). Verbal feedback was the preferred format, followed by written feedback and appraisal-based discussions (6). Most trainees preferred feedback to be delivered either immediately after patient encounters or at the end of clinical sessions (6). Trainees generally disliked receiving critical feedback in front of patients because of concerns regarding patient confidence and professional credibility (6).

The preferred feedback provider was a supervisor familiar with the trainee’s work and invested in their development (6). This finding reflects wider educational research demonstrating the importance of trust and supportive trainer–trainee relationships in effective feedback processes (6).

#### 3.5.3 Characteristics of effective feedback

Orthodontic trainees consistently identify clarity, specificity, actionability, and timeliness as key features of effective feedback (6). Studies also show that structured assessments incorporating feedback, such as Mini-CEX and OSCEs, improve clinical reasoning and learner confidence (4). Variability in assessor expectations and grading systems may reduce feedback consistency and educational value (4). Similarly, inconsistent feedback, overly critical assessments, or a lack of specificity can negatively impact confidence and engagement (4, 72).

Ramani and Krackov (73) summarized twelve suggestions for effective feedback, namely;

- Establishing a respectful learning environment,
- Communicating goals and objectives for feedback,
- Basing feedback on direct observation,
- Making feedback timely and a regular occurrence,
- Beginning the session with the learner’s self-assessment,
- Reinforcing good practice and highlighting necessary corrections,
- Using specific, neutral language to focus on performance,
- Confirming the learner’s understanding and facilitating acceptance,
- Concluding with an action plan,
- Reflecting on feedback giver skills,
- Creating staff development opportunities, and finally
- Making feedback part of institutional culture.

#### 3.5.4 Barriers to effective feedback

Time constraints represent the most reported barrier to effective feedback (6). Many trainees believe supervisors lack sufficient time to provide detailed discussion during busy clinical sessions (6). Additional barriers include discomfort receiving feedback publicly and concerns that supervisors may appear unapproachable (6). Resource limitations may also contribute to variability in feedback quality (4). Programs with stronger faculty support and technological infrastructure are often better able to provide structured assessments and regular feedback opportunities (4).

## 4 Recommendations and future directions

Although numerous ‘conceptual frameworks’ are applicable to medical education (80), only a selected few that are particularly relevant to orthodontic education are discussed here. Several recommendations emerge from the literature. First, postgraduate orthodontic programs would benefit from continued adoption of competency-based curricula aligned with international frameworks such as the WFO and NEBEOP guidelines (2, 13). Greater standardization may help reduce variability in graduate competence while preserving flexibility within different healthcare systems (2, 38, 74, 85–87). The UK training model (38), with its clearly defined curriculum domains, programmatic assessment structure, and intercollegiate examination system, provides a useful reference for international harmonization efforts. Structured assessment methods, including Mini-CEX, DOPS, OSCEs, and case-based discussions, are increasingly recognized as integral components of postgraduate training programs (4, 37).

The ultimate aim of postgraduate training programs is to cultivate sound clinical judgment and decision-making (84), a priority reflected in the growing presence of scenario-based assessment within exit examinations, such as the American Board of Orthodontics certification examination (75) and the UK dental specialty fellowship examinations (37). Further research into WBAs should specify that such research needs to test whether ‘deliberate practice principles’ (9, 10) are being met in current implementation, and not simply to measure trainee satisfaction.

Successful implementation depends on faculty development, assessor calibration, and consistent delivery of meaningful feedback (72).

Training programs may also benefit from fostering a stronger feedback culture (6). Allocating protected time for feedback (72), promoting supportive trainer–trainee relationships, and encouraging reflective practice may enhance both feedback quality and trainee engagement. In addition, technology-enhanced learning, PBL, and digital simulation can effectively supplement traditional teaching methods (4). Nevertheless, supervised patient care continues to represent a fundamental component of orthodontic education. Further longitudinal research remains necessary to evaluate how learning styles, educational strategies, and feedback systems influence long-term clinical competence and patient care outcomes (1, 4, 22).

## 5 Conclusion

Postgraduate orthodontic education has made meaningful progress through international frameworks, structured assessment tools, and increasingly active pedagogical approaches, but substantial variation in training quality and delivery persists worldwide. The evidence converges on a clear message: clinical competence develops through repeated, supervised, feedback-rich encounters aligned with the principles of deliberate practice, and current evidence suggests that digital innovations should complement rather than replace supervised clinical training. Trainees thrive when feedback is specific, timely, and delivered within supportive supervisory relationships. As the specialty continues to evolve, encompassing digital workflows, aligner therapy, and artificial intelligence, curricula must adapt accordingly, while preserving the irreplaceable core of supervised patient care. Rigorous longitudinal research remains the essential next step to link educational strategies with long-term clinical and patient outcomes.
